# Feasibility of collecting retrospective patient reported outcome measures (PROMs) in emergency hospital admissions

**DOI:** 10.1186/s41687-018-0077-y

**Published:** 2018-11-15

**Authors:** Esther Kwong, Nick Black

**Affiliations:** Department of Health Services Research, Faculty of Public Health and Policy, London School of Hygiene and Tropical Medicine, 15-17 Tavistock Place, London, WC1H 9SH UK

**Keywords:** Patient reported outcome measures, Health status, Health-related quality of life, Retrospective, Feasibility, emergency admissions, STEMI, emergency laparotomy

## Abstract

**Introduction:**

Outcome of emergency admissions is usually limited to mortality with little attempt to capture the views of health status of survivors. This is because of the challenge of determining patient reported outcome measures (PROMs) for the period before their emergency admission. The aim was to assess the feasibility of collecting retrospective PROMs to capture the pre-admission health status of patients admitted as emergencies.

**Methods:**

Prospective study of two cohorts: patients undergoing primary coronary angioplasty for acute ST elevation myocardial infarction (STEMI) in five hospitals and emergency laparotomy (EL) for gastro-intestinal conditions in 11 hospitals. Three rates were calculated: proportion of patients eligible for inclusion; proportion of eligible patients invited to participate; proportion of invitees who participated. Staff views were thematically analysed to understand factors that affected recruitment.

**Results:**

About 85% of patients were eligible of whom most were invited to participate (84% EL; 79% STEMI). The proportions of invitees agreeing to participate differed between STEMI (92%) and EL (72%), probably reflecting greater post-intervention morbidity in the latter.

Variation between hospitals was observed in the proportion deemed eligible (EL 72–97%; STEMI 63–100%), proportion invited (EL 60–93%; STEMI 71–96%) and the proportion of invitees agreeing to participate (EL 55–92%; STEMI 67–100%). While this might reflect case-mix differences between hospitals, it suggests there is scope for less well performing hospitals to improve their recruitment processes.

The extent to which this initial feasibility study was able to assess selection bias was limited to the age and sex of patients. There was no bias evident for EL patients but for STEMI, younger men were more likely to participate.

**Conclusion:**

It appears to be feasible to collect retrospective PROMs from patients admitted unexpectedly as emergencies for the two conditions studied. The relevance of these findings to other causes of emergency admissions needs to be established. In addition, these findings justify the case for a large, multi-site study that could explore unresolved concerns about selection bias, particularly those arising from the clinical characteristics of patients. It would also enable estimates of the extent of variation in PROMs between hospitals to determine the usefulness of using PROMs in emergency admissions.

## Introduction

In England, emergencies account for about 40% of all hospital admissions, with the number of admissions having increased by 47% over the last 15 years. Two-thirds of hospital beds are occupied by people admitted as emergencies and the cost is approximately £12.5 billion annually [1]. There is concern about variations in outcomes between providers [2–4]. While quite reasonably this has largely focused on mortality, there is also a need to consider outcome in terms of the health status of those who survive. To date, few attempts have been made to use patient reported outcome measures (PROMs) to determine patients’ perception of any change in their health status. As the aim of healthcare is to restore a patient’s health to his or her full potential, it is desirable to be able to compare patients’ outcome with their health status before the sudden and unexpected event that led to an emergency admission. The use of PROMs would enable clinicians to review the impact of their care on individual patients and allow organisations, including regulators, to assess and compare the outcomes of different providers.

Using PROMs in emergency admissions presents the methodological challenge of how to capture a pre-event measure for such patients as pre-existing data are, inevitably, not available. A recent literature review [5] found strong agreement in elective patients between the PROM they reported before admission with their later recall of that pre-admission health status (via a retrospective PROM). This has been confirmed in England in a recent study of elective surgical patients [6]. These findings suggest that a retrospective PROM can provide a means of obtaining baseline health status in the absence of a prospectively collected contemporary report. Assuming this is also true for emergency patients (something that inevitably can never be established through direct testing), it is important to know whether it would be feasible to collect retrospective PROMs in such patients and the optimal methods for achieving this.

Feasibility might differ from the situation with elective admissions because, unlike elective admissions, emergency patients are acutely unwell and may be distressed. In addition, the immediate clinical priority is their surgical or medical assessment and intervention. Thus, it would not be possible to collect a PROM until after initial treatment, during their recovery period some days later. Feasibility may also be influenced by the mode of administration and questionnaire design.

Only three studies have reported recruitment rates when using retrospective PROMs following emergency admissions. Two focused on trauma cases and one on acute lung injury. Gabbe and colleagues achieved 50% recruitment in trauma patients in two major Australian hospitals during their inpatient stay but boosted this to 77% by contacting them afterwards at home by mail and telephone [7]. Toien and colleagues who sought consent while trauma patients were in hospital in Norway and then surveyed them by mail afterwards achieved 50% recruitment [8]. And Gifford and colleagues reported 70% recruitment among survivors of acute lung injury in four major hospitals in USA [9].

The aim of this exploratory study was to assess the feasibility of capturing retrospective PROMs in emergency admissions for a common medical (primary coronary angioplasty for acute ST elevation myocardial infarction) and surgical (emergency laparotomy for gastro-intestinal system) reason in a representative sample of NHS hospitals. The primary objectives were to explore the three stages of recruitment: the proportion of emergency admissions that were eligible for inclusion; the proportion of eligible patients who were invited to participate by staff; and the proportion of patients invited who participated. The secondary objectives were: to determine the representativeness of recruited patients as regards their age and sex; and to compare recruitment rates in different hospitals to determine the potential maximum rate obtainable and the associated organisational factors.

## Methods

### Choice of conditions

The two clinical conditions were selected as both are the subject of a national clinical audit which aims to collect detailed clinical data from all cases. The National Emergency Laparotomy Audit (NELA) includes all patients over the age of 17 years undergoing an emergency laparotomy for gastrointestinal conditions in NHS hospitals in England and Wales [10]. The Myocardial Ischaemia National Audit Project (MINAP) collects data on all patients with acute ST elevation myocardial infarction (STEMI) who undergo a primary coronary angioplasty [11, 12]. Patients who met the national clinical audit criteria and were alive at discharge were considered for inclusion in this study. Patients were excluded if: they were not literate in English; judged not to have sufficient cognitive ability; or were not resident in the UK.

### Design

A multi-site study was carried out to ensure there would be some variation in the detailed organisation of patient recruitment and data collection. This would allow us to gain insights into the relative merits of recruiting in different settings and with different personnel involved [13]. For emergency laparotomy, 14 hospitals were selected on the basis of their high case ascertainment rates in NELA of which 13 agreed to participate. For STEMI patients, five primary angioplasty centres in London and the surrounding area were invited and all participated.

Sites were asked to recruit all eligible patients during a 15 week period. The study received ethics approval from South East Coast - Brighton & Sussex Research Ethics Committee (REC reference: 16/LO/2053) and it was incorporated in the NIHR Research Network Portfolio. Each site nominated someone to be the site lead (usually a consultant or senior research director) responsible for overseeing local data collection. Site leads then nominated study leads who undertook the data collection and liaised directly with one of us (EK) if any queries arose, completed a study log (see below), stored and returned the data. At some sites the site lead and study lead were the same person. Study leads at each site could delegate recruitment to appropriate members of the clinical team so the number of staff involved could vary.

### Patient recruitment

Staff were provided with training in the form of video clips and written materials. These materials were developed by EK from prior experience of collecting a retrospective PROMs for elective patients in two cohort studies [14]. Video materials were produced with the support of the University media team and research partners from the earlier study. EK also visited or held a telephone conference with staff at each site prior to the start of data collection (Appendix: Study Flow diagrams).

Patients were invited to participate once emergency medical and surgical treatments had been completed and as close to the discharge date as possible to ensure the immediate effects of the intervention (such as a general anaesthetic) were minimised. Clinical staff explained the study to patients and provided written information. Written consent was obtained from participating patients. Staff added a sticky label which included patients’ NHS numbers and some socio-demographic data (date of birth, sex, address). A questionnaire was completed by recruited patients once during their inpatient stay. Those impeded by physical disability or sensory impairment could be assisted by staff or family members reading aloud the questions and/or recorded responses on the questionnaire. They were cautioned to avoid influencing the patients’ views.

### Study log

Each study lead was required to complete a log covering every patient who met the national clinical audit criteria during the recruitment period. Staff recorded whether a patient met the eligibility criteria for inclusion in the study and if they were invited to participate. The date of consent of participants was also recorded. Patients’ reasons for declining to participate were recorded if an explanation was offered without direct questioning.

### Questionnaires

The questionnaires (paper hardcopy) included demographic information, self-reported co-morbidities, a disease-specific PROM and a generic PROM. The questionnaires contained instructions asking patients to recall how they were 1 month before their current admission. A systematic review identified suitable PROMs with adequate psychometric properties. Clinicians were then consulted in an unstructured meeting (a formal consensus development method was not used) to determine the final choice. This included consideration of the length and likely burden on patients of instruments.

For emergency laparotomy, the Gastro-Intestinal Quality of Life Index (GIQLI), developed by Eypasch and colleagues was selected [15]. It consists of 36 questions relating to the gastrointestinal system and the impact of symptoms and treatment on individuals’ physical, emotional and social status. It takes 5–10 min to complete and has good test-retest reliability (intra-class correlation coefficient = 0.92), and internal consistency (Cronbach’s alpha > 0.90). The GIQLI is the most commonly used validated PROM in studies investigating outcomes in emergency abdominal surgery [16].

For STEMI, the Seattle Angina Questionnaire (SAQ-7) is a 7 item health status measure for patients with coronary artery disease that has well-established validity, reliability, sensitivity to clinical change, and prognostic value [17, 18]. Scores range from 0 to 100, where higher scores indicate fewer symptoms and higher health-related quality of life. SAQ-7 has good domain coverage (symptom burden, functional status, and quality of life), psychometric properties (validity, sensitivity), feasibility to implement (questionnaire length, language availability, and cost to implement), and clinical interpretability (knowledge of how to interpret scores in a clinically meaningful way) [19].

Both groups completed a generic PROM, the EQ-5D-3 L. This has five items concerning the domains of mobility, usual activities, personal care, pain/discomfort and anxiety/depression. It takes up to 5 minutes to complete [20]. For each of these questions, the respondent chooses from three responses indicating the level of their function. A multi-attribute utility score where death and perfect health are represented by 0 and 1 are calculated [21]. Scores less than 0 are considered worse than death and 1 is the maximum score possible. The EQ-5D-3 L was used rather than the EQ-5D-5 L as the former is still the version used in the National PROMs Programme in England.

### Analysis

#### Quantitative analysis

For each condition, three rates were calculated: the proportion of all admissions with the condition that staff considered met the eligibility criteria for inclusion; the proportion of eligible patients invited to participate by staff; and the proportion invited who participated. In addition, the representativeness of those participating was assessed by comparison with all those included in the national clinical audit, though this was only possible for age and sex as clinical data were not available. The performance of hospital sites was compared to establish the maximum possible rates that could be obtained.

#### Qualitative analysis

At the completion of the study, information was sought from the site leads using a structured form administered by telephone interview or email. These observations, supplemented by a field diary kept by EK, were subjected to thematic analysis to identify the factors that facilitated and impaired patient participation to learn how data collection might be maximised.

## Results

### Quantitative results

#### Emergency laparotomy

Of the 13 hospitals that agreed to participate, 11 collected data for the full 15 week duration of the study. Two hospitals stopped after 1 month due to local staff changes and their data are not included in the analyses. Those two hospitals were the only ones where the site lead was a non-clinical audit manager.

In all 11 participating hospitals the site lead was either a consultant surgeon or anaesthetist. They took responsibility for identifying patients from their NELA database and ward lists and provided oversight of the data collection. In nine sites the study lead was a nurse (usually a research portfolio nurse). They invited and consented patients, and undertook the data collection on weekdays. In the other two sites doctors took on these tasks. Some sites had additional staff to support managing the study log, arranging paperwork and covering periods of leave.

**Fig. 1:**
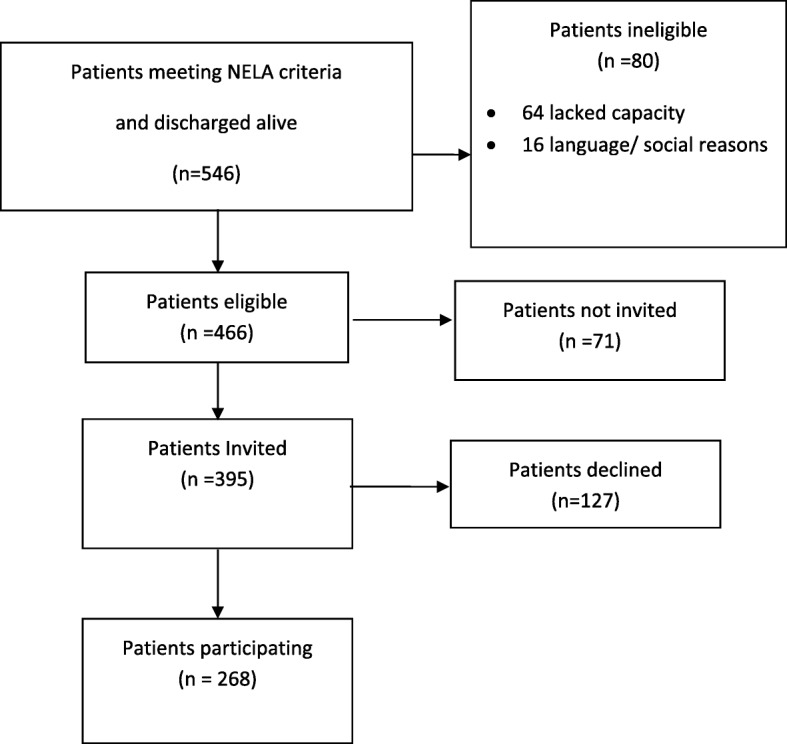
Recruitment Flow Diagram for Emergency Laparotomy patients

During the recruitment period, 546 emergency laparotomy patients were admitted and survived to discharge, of which 466 (85%) were deemed eligible to participate (Fig. 1). Of the 80 ineligible patients, 64 were considered to lack capacity to consent and complete a PROM and 16 were not literate in English.

Of the 466 eligible patients, 395 (85%) were invited to participate. The main reasons for not inviting patients was that the patient was discharged rapidly (e.g. transfer to another hospital, self-discharge) or discharged at weekends when staff collecting data were not at work.

Of the 395 invited, 268 (72%) patients agreed to participate and completed a questionnaire. Of the 127 who declined to participate, the most common reason recorded by staff was that they were feeling too tired to complete the questionnaire.

**Fig. 2:**
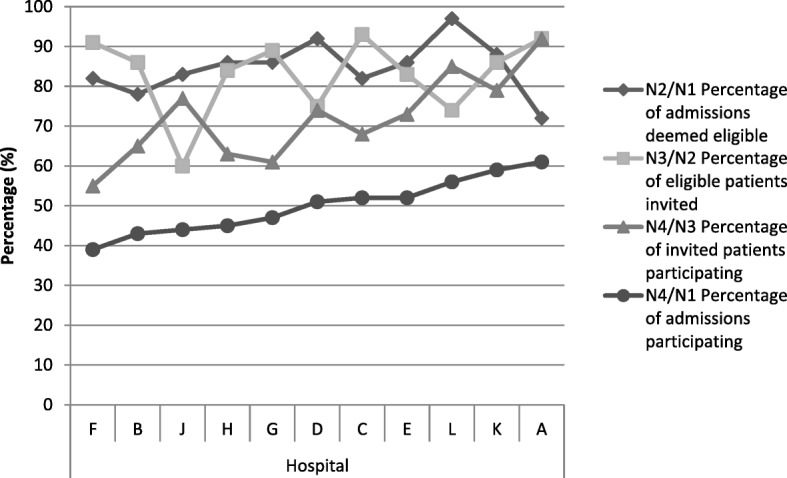
Relationship between the proportions of emergency laparotomy patients recruited at each of the three stages, by hospital

There was some variation across the 11 sites. The proportion deemed eligible ranged from 72 to 97%, those invited from 60 to 93% and those agreeing to participate from 55 to 92% (Table 1). There was no consistent relationship between the three rates (Fig. 2). Causes of low overall recruitment could be because eligible patients were not invited (J) or patients declined such invitations (F). Those with the highest overall participation included the hospital with the highest proportion deemed eligible (L) and the one with the lowest eligible proportion (A).

**Table 1:** Emergency Laparotomy recruitment overall and by hospital (*n* = 11)

	Hospital											
	A	B	C	D	E	F	G	H	J	K	L	Overall
N1 Number of admissions discharged alive	18	46	81	39	21	54	64	110	18	56	39	546
N2 Number of eligible patients	13	36	67	36	18	44	55	95	15	49	38	466
N3 Number invited to take part	12	31	62	27	15	40	49	80	9	42	28	395
N4 Number participated	11	20	42	20	11	21	30	50	8	33	22	268
N2/N1 Percentage of admissions deemed eligible	72	78	82	92	86	82	86	86	83	88	97	85
N3/N2 Percentage of eligible patients invited	92	86	93	75	83	91	89	84	60	86	74	85
N4/N2 Percentage of eligible patients participating.	85	56	63	56	61	47	55	53	48	67	59	59
N4/N3 Percentage of invited patients participating.	92	65	68	74	73	55	61	63	77	79	85	72
N4/N1 Percentage of admissions participating	61	43	52	51	52	39	47	45	44	59	56	49

Patients who participated were representative of all admissions as regards sex (male 47% v 48%) and age (median 66 v 67 years) [10].

#### Primary angioplasty for STEMI

All five sites participated for the full study duration of 15 weeks. The site leads in four hospitals were the hospital or cardiology department research manager or director. In the other hospital a nurse consultant was site lead. The study lead responsible for recruiting patients and collecting data during weekdays in all sites was a nurse (primarily research portfolio nurses, with the support of ward nurses). Some sites had additional administrative research staff to support managing the study log and arranging paperwork.

**Fig. 3:**
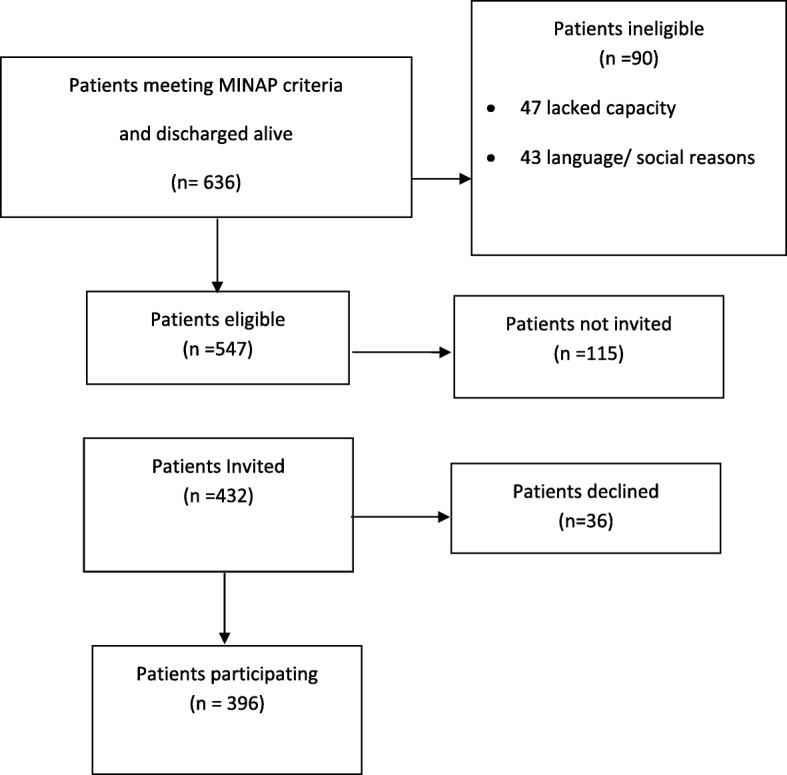
Recruitment Flow Diagram for STEMI patients

A total of 636 ST-elevation myocardial infarction patients meeting the MINAP criteria were admitted during the 15 week study period and survived to discharge (Fig. 3). 547 patients (86%) met the study’s inclusion criteria and were eligible for invitation. Ineligible patients included 47 who lacked sufficient cognitive capacity, 36 not literate in English and 7 had no UK residence.

Of the 547 eligible to participate, 432 (79%) were invited by staff to participate. The main reasons for not inviting patients was that the patient was discharged rapidly (e.g. transfer to another hospital, self-discharge) and those discharged at weekends or at night when staff collecting data were not at work. Of the 432 invited, 396 (92%) patients participated and completed a questionnaire. Of the 36 who declined to participate, most provided no reason.

**Fig. 4:**
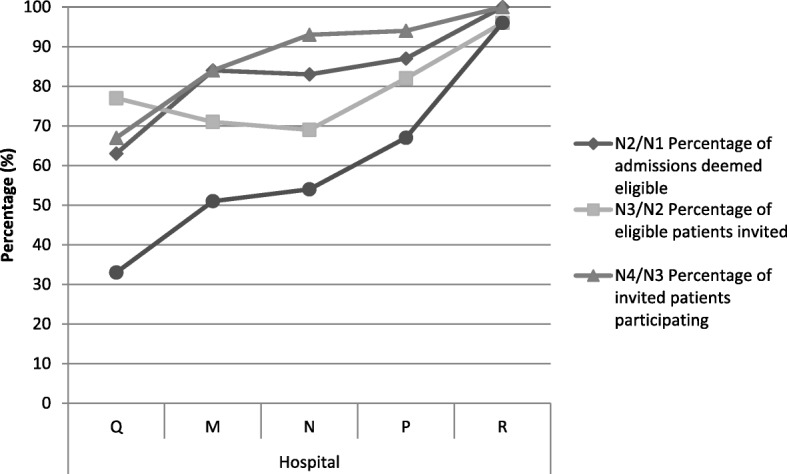
Relationship between the proportions of STEMI patients recruited at each of the three stages, by hospital

There was some variation across the five sites. The proportion deemed eligible ranged from 63 to 100%, those invited from 69 to 96% and those agreeing to participate from 67 to 100% (Table 2). Unlike with EL, there was some consistency in the relationship between the rates for the three stages (Fig. 4). In hospital Q with the lowest recruitment proportion (33%), the rates were poor for all three stages. In contrast, hospital R with the highest proportion recruited (96%) achieved this by success in all three stages.

**Table 2:** STEMI recruitment overall and by hospital (*n* = 5)

	Hospital					
	M	N	P	Q	R	Overall
N1 Number of admissions discharged alive	180	156	123	49	128	636
N2 Number of eligible patients	152	129	107	31	128	547
N3 Number invited to take part	108	89	88	24	123	432
N4 Number participated	91	83	83	16	123	396
N2/N1 Percentage of admissions deemed eligible	84	83	87	63	100	86
N3/N2 Percentage of eligible patients invited	71	69	82	77	96	79
N4/N2 Percentage of eligible patients participating.	60	65	78	52	96	72
N4/N3 Percentage of invited patients participating.	84	93	94	67	100	92
N4/N1 Percentage of admissions participating	51	54	67	33	96	62

Patients who participated were more likely to be male (79% v 72%) and slightly younger (median: males 60 v 63 years; females 67 v 71) than all those included in the national clinical audit [10].

### Qualitative results

Staff identified facilitators and obstacles at each stage of recruitment. .

#### Identification of eligible patients

Staff found identification of eligible EL patients easier if the site lead was also involved in the national clinical audit. Identification was facilitated by combining their NELA register, emergency theatre lists and consultants’ knowledge of patients. This was easiest in sites with a real time NELA register and electronic patient trackers. Similarly, for STEMI identification was aided by the existence of pathway activation records. Conversely, for EL the relocation of patients (such as from ITU to ward) could delay identification as a patient could be temporarily ‘lost’. This was rarely a problem for STEMI as patients were admitted to a designated ward or coronary care unit and rarely moved to other locations.

#### Inviting patients to participate

Timely identification of patients and their location was crucial to enable study leads to invite patients. The main reason that patients were not invited was because of missing the target period of 1–2 days before discharge. This was a particular problem at weekends. As many STEMI admissions stayed less than 48 h, patients admitted on a Friday would be discharged over the weekend and thus risk not being invited as study leads were not available. The site that managed to capture all patients (R) did not routinely discharge patients over the weekend. One proposed solution is to involve members of the ‘on-call’ clinical team at weekends.

An additional challenge with EL patients was predicting when this window of opportunity would occur or when discharge would occur as there was greater variation between patients. One way of coping with this with EL patients was for staff to invite them as soon as they felt there was an opportunity to speak to them, such as after stepping down from ITU to the ward.

#### Gaining agreement from patients to participate

Staff felt that patient participation was more likely if they were approached in an open and positive manner, explaining the purpose of the study clearly. Also, bringing in members of the clinical team directly involved in their care helped.

Patients attitudes about the reasons for PROMs, their health status and the extent to which they had come to terms with their emergency admission were factors that affected their agreement to participate. Patients understood and welcomed the value of PROMs when their purpose was explained by engaged staff.

Most patients were glad to be asked for their views. The perceived time involved affected some decisions. STEMI patients welcomed the brevity of the questionnaire and while some EL patients were initially perceived the questionnaire to be too long, once they had seen that the questions were straightforward to complete (closed rather than open), most agreed to participate.

The main reason patients declined was they did not feel well enough to complete a questionnaire. Acceptance was greater once patients had had time to come to terms with the significant medical events they had experienced. As staff, for ethical reasons, were not able to revisit a declined invitation when a patient felt better, there was a delicate balance needed between avoiding decline and missing the opportunity, such that they were discharged home already. Given that the speed of recovery varied between patients, it was difficult to make the right judgment. Staff tried to invite as close to discharge as possible even if that risked missing patients.

## Discussion

### Main findings

Patients can successfully be recruited to complete PROMs during their inpatient admission following significant emergency treatment (primary angioplasty and emergency laparotomy). Identification of relevant patients presented few difficulties, partly because the patients were also being included in a national clinical audit. It may prove to be more problematic if no such audit existed.

Of those patients admitted, 86% met the eligibility criteria to be invited to complete a PROM questionnaire about their pre-admission health status. Most of those deemed eligible were invited (85% emergency laparotomy; 79% STEMI). The main reasons for not inviting patients was that the patient was discharged rapidly (e.g. transfer to another hospital, self-discharge) or at weekends or out-of-hours when staff collecting data were not at work.

Agreement by patients to participate differed between the two conditions: 92% for STEMI patients but only 72% for emergency laparotomy. This probably reflected the greater post-intervention morbidity of the latter group. Despite the modest participation rate of laparotomy patients, they were representative of all such patients as regards age and sex, though might have differed in other respects (clinical severity, comorbidities etc). The observation that STEMI participants were more likely to be male and younger is of little concern given the very high participation rate among this group.

There was variation in the eligibility, invitation and participation rates between the hospitals. While some of this might reflect case-mix differences between hospitals (e.g. English literacy), these differences suggest that there is scope for less well performing hospitals to improve their recruitment processes. The reason for low eligibility in some sites - 72% for laparotomy in hospital A and 63% for STEMI in hospital Q - requires investigation to see if these rates are clinically justified. Similarly, low invitation rates (62% in hospital J and 69% in hospital N) suggest that overall recruitment could be enhanced given that other sites achieved invitation rates of over 90%.

The lower proportions of patients agreeing to participate in some hospitals (55% in hospital F for laparotomy and 67% in hospital Q for STEMI) may reflect case-mix differences but it might be because staff were less enthusiastic and effective in how they approached and invited patients.

### Comparison with other studies

This is the first study in England to demonstrate the feasibility of collecting retrospective PROMs in emergency hospital admissions. The only previous studies to report on recruitment rates of emergency admissions involved either trauma patients [7, 8] or critical care survivors (not all of whom were emergency admissions to hospital) [9]. Despite all three studies being concentrated in only 1–4 sites, in our multi-site study we achieved similar proportions of admissions participating (49% and 62%) to those previously reported (50–77%).

### Strengths and limitations

Its strengths are that it considered both a common medical and surgical reason for an emergency admission, included both a disease-specific and generic PROM that varied in length, and we observed the recruitment performance in a wide range of 16 hospitals which differed in terms of annual volume of cases, teaching status and geographical location.

Despite this, some caution is needed in interpreting the generalisability of the results. First, the hospitals that participated in the emergency laparotomy study were those that were achieving a high case ascertainment rate in the national clinical audit so may have characteristics and a culture that is more likely to support the collection of PROMs. As regards the STEMI sites, all five were located in and around London (for greater ease of access for the research team) so may differ from other parts of the country. This may explain why patients were slightly younger than that seen nationally.

Second, it is possible that the response of staff and patients to collecting PROMs for the two clinical conditions selected might not be replicated with other reasons for emergency admission. This will need to be investigated in subsequent implementation of retrospective PROM collection.

Third, about 14% of patients were excluded from this study as ineligible (those not literate in English and those with cognitive impairment). The proportion excluded varied between hospitalsfor EL (3–21%) and for STEMI (0–37%). Given that such differences could introduce some selection bias, future comparisons of hospitals’ outcomes could be undermined if this was not taken into account. Investigations are needed to establish if such differences reflect the populations being served or the perceptions of staff as to the ability of patients to participate. In addition, recruitment of people not literate in English might be increased with the provision of questionnaires in other languages or translation services. For patients with cognitive impairment, the use of proxy-reported PROMs should be investigated.

Fourth, the extent of any selection bias was limited by the lack of data on patients’ clinical characteristics. A further feasibility study with larger samples of patients, linked to their clinical characteristics, is needed so sub-group analyses could quantify the extent of any selection bias. This would also permit investigation of any desirability bias affecting which patients agree to participate.

Finally, given that the only means of obtaining patient reported health status in emergency admissions is by the use of retrospective PROMs, there will always be some uncertainty as to the impact of recall bias and response shift. However, unless it is believed that these biases differ systematically between hospitals, there is little risk to the meaningfulness of hospital comparisons.

### Implications for practice

While the overall rates of eligibility, invitation and participation were good, they could be improved if those hospitals with lower rates adopted some of the processes that higher performing hospitals used. There are several potential ways of increasing recruitment in all three stages:

*Support timely identification of patients*

- integrate PROMs into the collection of data for the national clinical audit
- automatic PROM reminders as part of national clinical audit

*Improving timing of invitation*

- encourage the research nurses to participate in ward rounds to increase the support of ward nurses
- involve other clinical staff at weekends (if discharges fall on this day)

*Improving staff ability to invite patients*

- engage all relevant clinical staff to ensure the aim and purpose of collecting recalled PROMs is understood
- embed PROMs collection with the completion of discharge forms
- reduce staff workload by simplifying the patient information sheets and consenting procedure.

*Increasing patients’ acceptance*

- invite patients to participate as close to the discharge date as possible

## Conclusions

This initial feasibility study has suggested that it is feasible to collect retrospective PROMs from patients admitted unexpectedly as emergencies for the two conditions studied across a variety of types of hospitals in the NHS. The relevance of these findings to other causes of emergency admissions needs to be established. In addition, these findings justify the case for a large, multi-site study that includes clinical information on participants could explore the unresolved concerns about selection bias, particularly those arising from the clinical characteristics of patients. It would also enable estimates of the extent of variation in PROMs between hospitals to determine the usefulness of using PROMs in emergency admissions.

## Figures and Tables

**Fig. 5:**
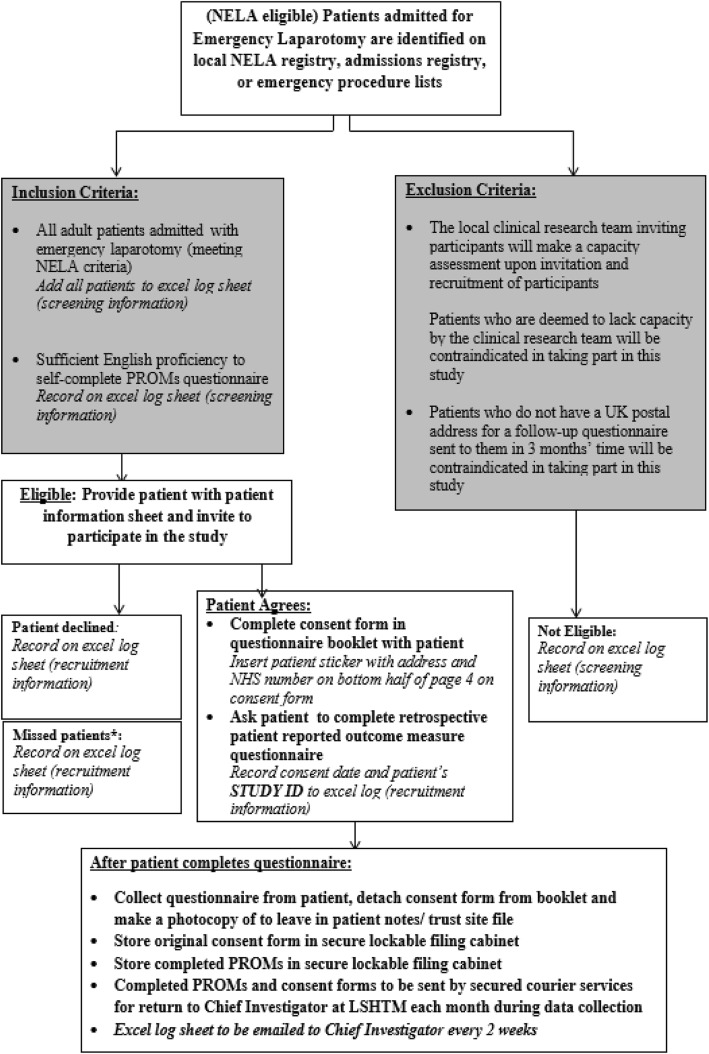
Emergency Laparotomy Study Flow Diagram

**Fig. 6:**
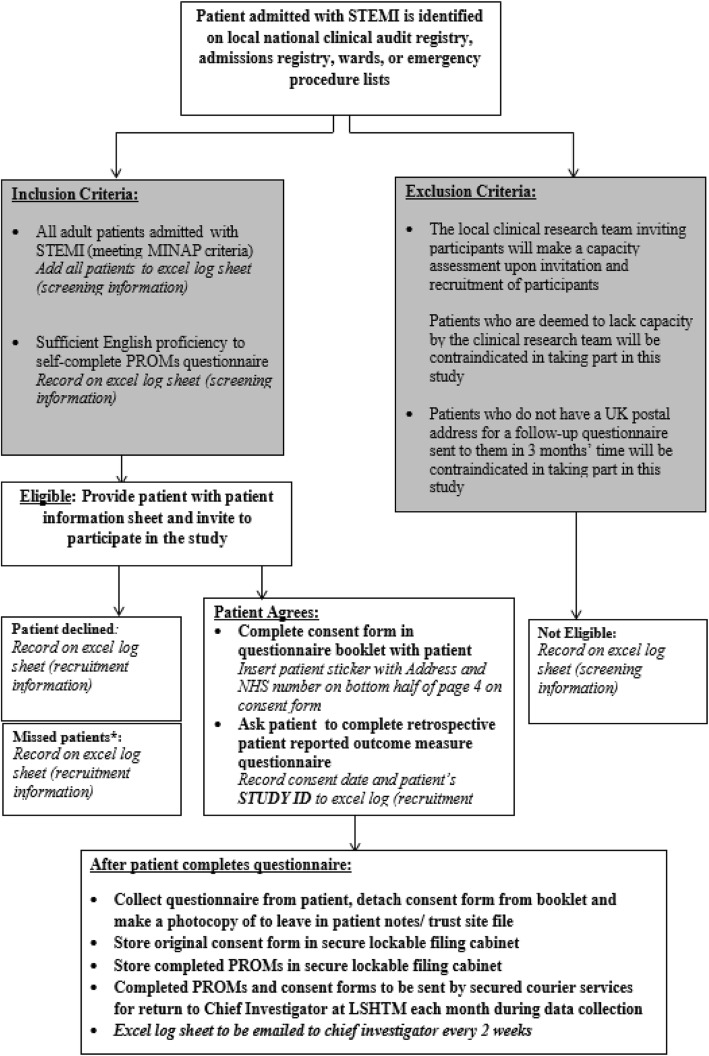
STEMI Study Flow Diagram
